# Immunization Treatment

**Published:** 1923-05

**Authors:** 


### IMMUNIZATION TREATMENT

### THE PRIMARY CAUSE

Surprisingly little is definitely known of the primary cause of colds. There is a wide-spread belief that some of the common bacteria found in the nose and throat stand in causative relation to this condition. Others consider their presence too variable and inconstant to warrant any conclusion that they are the primary cause, and find the most convincing evidence to favor a filterable virus.[1]

The general practitioner knows, however, that the primary disease, whether caused by a filterable virus or not, is manifested, first, by hyperemic phenomena in the upper air passages, with serous discharge, followed by a very pronounced increase in the number and activity of the bacterial flora. He knows also that the local complications, including sinus infections, middle ear infections, laryngitis, tracheitis, bronchitis and, rarely, pneumonia and sepsis, are chiefly due to the pyogenic organisms accompanying the "cold."

### PREVENTION

Not only the cause of colds, but also the value of bacterins for preventive immunization, has been widely discussed among experts and specialists. Some of the most recent writers, including Eyre and Lowe,[2] Von Sholly and Parke,[3] present the results of controlled experiments which indicate that the vaccines have beneficial influence. Leading immunologists of high standing, *e. g.*, Fleming & Clemenger,[4] definitely state that "the duration of the purulent discharge can be diminished, and the individual protected against the infection."

General practitioners are using this method in their daily practice and are accumulating practical clinical ex-

1 Bloomfield, Bul. Johns Hopkins Hospital 32; 12, April, 1921.
2 Eyre & Lowe, The Lancet, October 12, 1918, p. 484.
3 Von Sholly & Parks, Med. Record, Dec. 11, 1920, p. 1004.
4 Fleming & Clemenger, Nelson's Loose Leaf Med., Vol. I, 1920.

periences which, if they could all be brought together, would offer most convincing affirmative testimony.

A very large number of unpublished reports and verbal communications, testifying to the benefit derived from the regular use of prophylactic immunization, have reached us. In general they parallel closely the published statement of Whitacre,[5] who says:

"My own experience with vaccines in colds has been very convincing. For some years I had been subjected to severe bronchitis each winter. Four years ago I had an attack which necessitated my going to the hospital and the services of a private nurse. I had vaccine administered and was back at work after the tenth day. I am convinced that with ordinary therapy in such a condition I should have been sick much longer and at least fared no better. Each fall I have taken prophylactic inoculations, last winter having had only one. As I have been free from coryza or bronchitis, I feel justified in believing I carry a higher immunity than I formerly did to the organisms causing common colds and bronchitis.

*"When former patients come back to my office and ask for vaccine treatment for themselves and their friends against the effects of common colds and allied catarrhal conditions, season after season, and never think of taking or asking for medicines to relieve and protect themselves against these common ailments, I am thoroughly convinced that they have derived more benefit from this method than from any other they had received for this trouble. People do not willingly subject themselves to the unpleasantness of hypodermic treatment unless they are very sure there is nothing else that will take its place."*

### WHAT PRODUCT TO USE

The important work of Allen on the bacteriology of common colds was supplemented by similar studies conducted by experts in the Mulford Laboratories and led to

[5] Whitacre, International Jourl. of Surg., July, 1922, p. 258.

the introduction of a bacterial vaccine containing organisms commonly found in a large percentage of cases. This was followed later by the introduction of a *sensitized* bacterial vaccine, as an addition to our list of serobacterins.

Some twelve years experience confirms our confidence in "Catarrh-Cold Serobacterin Mixed." Physicians will find it the best available product for immunizing purposes. It is a sensitized bacterial vaccine, containing strains of the principal organisms commonly found in respiratory infections. It is supplied in syringes, or vials, of the following counts per cc.

| Formula | A | B | C | D | Doses |
|---|---|---|---|---|---|
| Staphylococcus aureus ............ | 125 | 250 | 500 | 1000 | million |
| Staphylococcus albus ............. | 125 | 250 | 500 | 1000 | million |
| Streptococcus (hemolytic and non-hemolytic) ........'.......... | 125 | 250 | 500 | 1000 | million |
| Pnemococcus (Types 1, 11 and 111) | 125 | 250 | 500 | 1000 | million |
| M. Catarrhalis (group)........... | 125 | 250 | 500 | 1000 | million |
| B. Friedlander ................... | 125 | 250 | 500 | 1000 | million |

Those physicians who desire to establish some immunity against the *influenza bacillus,* in addition to those mentioned in the above formula, should order "Influenza Serobacterin Mixed," another well-known sensitized catarrhal vaccine, which the Mulford Laboratories have supplied for a number of years.

### HOW TO USE IT

The common practice with average individuals is to administer one series of immunizing doses (A. B. C. and D.) in the fall or early winter, with the result that satisfactory protection lasts throughout the season. For particularly susceptible persons, some physicians recommend that they be immunized early in the fall (A. B. C. and D. doses) and that their immunity be maintained by injecting the *D dose* at ninety day intervals thereafter.

The formula given above represents the usual preventive dosage, and the Serobacterin (sensitized vaccine) may be administered at intervals as short as 48 hours, but a longer interval between doses is perhaps preferable. Clinical symptoms observed by the physician following the initial dose will be a valuable guide in making such modifications in the size and intervals of subsequent doses as may be necessary. In children, the doses should be reduced in proportion to age, administering one-tenth to one-half the adult dosage.

The serobacterin is preferred because it is a combination of antibodies and antigens which confers a degree of *immediate* passive resistance to invading organisms, and in its preparation the bacteria are markedly reduced in toxicity. Consequently its use is followed by less tendency to severe local or systemic reactions and permits an administration of larger doses at more frequent intervals.

Serobacterins consist of *antibodies* held in loose combination with the *antigens* (bacteria) and, when injected, some of this antibody charge is set free, imparting *immediately* a degree of protection, passive in character which persists until the tissue cells have been stimulated to the production of antibodies which impart active immunity.

